# Quantification of left ventricular functional parameter values using 3D spiral bSSFP and through-time Non-Cartesian GRAPPA

**DOI:** 10.1186/s12968-014-0065-1

**Published:** 2014-09-11

**Authors:** Kestutis J Barkauskas, Prabhakar Rajiah, Ravi Ashwath, Jesse I Hamilton, Yong Chen, Dan Ma, Katherine L Wright, Vikas Gulani, Mark A Griswold, Nicole Seiberlich

**Affiliations:** Biomedical Engineering, Case Western Reserve University, Cleveland, Ohio USA; Cardiothoracic Imaging, Department of Radiology, University Hospitals Case Medical Center, Cleveland, Ohio USA; Pediatric Cardiology, Rainbow Babies and Children’s Hospital, University Hospitals Case Medical Center, Cleveland, Ohio USA; Radiology, University Hospitals Case Medical Center, Cleveland, Ohio USA

**Keywords:** Left ventricular functional CMR, Spiral, GRAPPA, Non-Cartesian, Parallel imaging

## Abstract

**Background:**

The standard clinical acquisition for left ventricular functional parameter analysis with cardiovascular magnetic resonance (CMR) uses a multi-breathhold multi-slice segmented balanced SSFP sequence. Performing multiple long breathholds in quick succession for ventricular coverage in the short-axis orientation can lead to fatigue and is challenging in patients with severe cardiac or respiratory disorders. This study combines the encoding efficiency of a six-fold undersampled 3D stack of spirals balanced SSFP sequence with 3D through-time spiral GRAPPA parallel imaging reconstruction. This 3D spiral method requires only one breathhold to collect the dynamic data.

**Methods:**

Ten healthy volunteers were recruited for imaging at 3 T. The 3D spiral technique was compared against 2D imaging in terms of systolic left ventricular functional parameter values (Bland-Altman plots), total scan time (Welch’s t-test) and qualitative image rating scores (Wilcoxon signed-rank test).

**Results:**

Systolic left ventricular functional values were not significantly different (i.e. 3D-2D) between the methods. The 95% confidence interval for ejection fraction was −0.1 ± 1.6% (mean ± 1.96*SD). The total scan time for the 3D spiral technique was 48 s, which included one breathhold with an average duration of 14 s for the dynamic scan, plus 34 s to collect the calibration data under free-breathing conditions. The 2D method required an average of 5min40s for the same coverage of the left ventricle. The difference between 3D and 2D image rating scores was significantly different from zero (Wilcoxon signed-rank test, p < 0.05); however, the scores were at least 3 (i.e. average) or higher for 3D spiral imaging.

**Conclusion:**

The 3D through-time spiral GRAPPA method demonstrated equivalent systolic left ventricular functional parameter values, required significantly less total scan time and yielded acceptable image quality with respect to the 2D segmented multi-breathhold standard in this study. Moreover, the 3D spiral technique used just one breathhold for dynamic imaging, which is anticipated to reduce patient fatigue as part of the complete cardiac examination in future studies that include patients.

**Electronic supplementary material:**

The online version of this article (doi:10.1186/s12968-014-0065-1) contains supplementary material, which is available to authorized users.

## Background

Cardiovascular magnetic resonance (CMR) is useful for assessing the structure and function of the left ventricle (LV) [1]. Since the data for each slice are acquired over multiple heartbeats with k-space segmentation [2], the patient must perform a breathhold to avoid respiratory motion artifacts. Typically, even when two or more slices can be collected within each breathhold, coverage of the entire LV in the short-axis orientation requires multiple breathholds. The unavoidable variations in diaphragm position with each breathhold can lead to slice-dependent shifting of the cardiac anatomy within the imaging volume [3], which has been shown to contribute to inter-examination variability in LV functional parameters [4]. As shown on a more recent 1.5 T MR scanner, reproducibility in patient populations has improved [5], where test-retest studies of ejection fraction and mass have been shown to be more variable for patients with congestive heart failure with respect to other conditions and healthy controls [6]. Furthermore, performing multiple breathholds in quick succession can lead to fatigue [7], especially in patients with poor cardiac and respiratory function, where hyperventilation with room air allowed an average breathhold duration of just 12 s [8]. Although introducing gaps between the slices can reduce the number of breath holds, small pathological abnormalities in the morphology and motion of the LV can be missed due to the resulting discontinuous coverage. In short, collecting all dynamic data for LV functional parameter analysis within a single breathhold would resolve the aforementioned challenges and shorten the overall scan time.

Single-breathhold 3D imaging methods have demonstrated equivalent left ventricular functional parameter values as the multi-slice approach without the confounding effects of variable diaphragm position. One of the earliest proposals combined three-fold SENSE acceleration in the phase encoding direction with partial Fourier acceleration in the partition encoding direction of a 3D Cartesian acquisition [9]. This method collected volumetric data over a breathhold of about 20 s, and interpolation was used to reconstruct timeframes at a temporal resolution of 40-53 ms. The acquired temporal resolution (or footprint), as defined as the amount of time within each heartbeat that was used to accumulate a portion of the k-space data for each cardiac phase image was 76 ms. A recent study showed that dynamic single-breathhold 3D Cartesian data could be encoded with 1.45 × 1.45 mm^2^ resolution at TR 3.5 ms, where SENSE R = 2×2 and 62% partial Fourier in the phase encoding direction reduced the breathhold to 18-25 s [10]. Additional strategies have been developed to accelerate 3D Cartesian encoding with high in-plane resolution and short TR for robust bSSFP cine imaging. For example, 3D k-t BLAST [11] encoded dynamic data along a sheared 3D Cartesian grid at 2 × 2 × 5 mm^3^ resolution and TR 3.1 ms; however, the breathhold of 25-27 s was long despite R = 5 acceleration, and the training data was acquired in a separate breathhold. The breathhold for the dynamic scan for 3D k-t BLAST was reduced to an average of 15 s (plus an additional breathhold of 7-14 s for training data) by increasing acceleration to R = 6 and encoding data with in-plane resolution near 3 × 3 mm^2^ followed by spatial interpolation [12]. Lower blood-myocardium contrast from reduced in-flow enhancement for 3D cine cardiac imaging [13] was improved for 3D k-t BLAST with the use of a contrast agent [14]; this study acquired dynamic data with a resolution of 2.4 × 2.4 × 10-12 mm^3^ and then interpolated to 1.2 × 1.2 × 5-6 mm^3^. However, Bland-Altman analysis of systolic LV functional parameters still showed significant differences with a 2D cine standard. Note that radial encoding also enables high resolution imaging using a short TR. For instance, a five-fold undersampled ECG-gated 3D radial stack of stars trajectory with zero-filling reconstruction and UNFOLD temporal filtering was used to generate 3D cine images from dynamic data with a temporal footprint of 71 ms with a breathhold of 24 RR intervals [15]. Other investigators introduced a 3D multi-echo radial trajectory [16], which obviated the need for angular undersampling and temporal filtering, where data were acquired at 1.3 × 1.3 × 8 mm^3^ spatial resolution and 45 ms temporal resolution in a single breathhold of 17 s. This method segmented the projection angle direction across ECG triggers, where the long TR (i.e. 4.5 ms) required both partial Fourier acceleration and a limited partition encoding matrix size to keep the temporal footprint of each cardiac phase under 50 ms. Finally, a Compressed Sensing reconstruction has been recently described for a 3D radial trajectory [17], where the promising images obtained from dynamic data that was acquired at R = 10.7 acceleration, 40.5 ms temporal resolution and 2.1 × 2.1 × 8 mm^3^ spatial resolution in a single breathhold of 27 s were not compared against a 2D cine standard. Note that several other rapid MR acquisition and reconstruction methods in a review article [18] could also be adapted for rapid 3D imaging of left ventricular motion. However, the 3D bSSFP cine method should meet as many of the following objectives as possible: a single, short breathhold for dynamic imaging; a short TR to minimize off-resonance effects; low parallel imaging acceleration factor; tolerance for motion in the calibration data (if needed); high in-plane resolution without spatial interpolation; high temporal resolution without filtering or interpolation; the freedom to adjust the partition encoding direction for coverage or resolution within the constraint of a single breathhold; and, a fast, linear and non-iterative reconstruction process.

This work uses the parallel imaging concept known as through-time non-Cartesian GRAPPA to rapidly acquire the data for left ventricular ejection fraction analysis. The first application of through-time non-Cartesian GRAPPA enabled single-slice free-breathing real-time CMR imaging by shortening the acquisition time of a 2D radial acquisition [19]. A free-breathing prescan was used to collect all projection angles multiple times in order to calibrate radial GRAPPA weights. These geometry-specific GRAPPA weights were then applied to the undersampled radial data to yield images with temporal resolutions of 30-50 ms. By substituting a variable density spiral for the radial trajectory, the required acceleration factor could be reduced while simultaneously improving the temporal resolution of the scan [20]. In contrast to many other rapid MRI techniques [18], neither temporal filtering nor sliding window reconstruction were needed to achieve short temporal footprints per cardiac phase, and the reconstruction is linear and non-iterative with well-known and predictable reconstruction artifacts. The through-time non-Cartesian GRAPPA technique was recently extended to accelerated 3D imaging, where an examination of the acquisition and reconstruction of undersampled 3D data was first shown for renal angiography with a 3D radial stack of stars sequence [21]. The work described here employs an accelerated 3D stack of spirals acquisition with 3D through-time spiral GRAPPA to enable the collection of 3D timeframes of left ventricular motion in a single breathhold.

This study compares the single-breathhold 3D spiral method with a 2D multi-breathhold standard. First, systolic left ventricular functional parameter values, namely end-diastolic volume, end-systolic volume, ejection fraction and end diastolic mass were analyzed for significant differences. Second, total scan time was evaluated. Finally, an image rating study was performed to quantify differences in image quality between the acquisition methods.

## Methods

Ten healthy volunteers provided written informed consent to participate in this study that was approved by the local Institutional Review Board. All imaging was performed on a 3.0 T MR scanner (MAGNETOM Skyra, Siemens Healthcare, Erlangen, Germany) using a body and spine array combination (up to 32 channels). Image reconstruction of the 3D scans was performed offline with Matlab (Version R2011b, Mathworks, Nattick, MA).

### Pulse sequences

An ECG-gated 2D segmented cine bSSFP sequence served as the gold standard for functional parameter and image quality comparisons. Key sequence parameters were: TE 1.43 ms, TR 3.26 ms, 12 slices, slice thickness 8 mm with no gap, acquired FOV 284×340 mm^2^, acquired matrix 144×208, GRAPPA R = 2 and readout bandwidth 960Hz/pixel. Note that the phase encoding direction was symmetrically zero-filled to 174 lines to yield pixels with numerical isotropic resolution. By using a segment size of 12 phase encoding steps, the temporal resolution was 39 ms per cardiac phase with an in-plane resolution of 1.63×1.63 mm^2^. The encoding of each slice required 6 RR intervals (due to GRAPPA R = 2) plus 1 RR interval to enter steady state. The data for each slice were collected in a single separate breathhold. Although non-accelerated multi-slice imaging with one slice per breathhold has been used by other investigators [22], GRAPPA R = 2 eliminated fatigue as a confounding factor in the gold-standard images in this study at the expense of scan time efficiency. Due to SAR constraints, the flip angle ranged from 40° to 62° across this set of volunteers. The total scan time to complete this sequence, including the duration for instructions and the breathhold and rest periods, was recorded for comparison against the proposed 3D method.

**Figure 1:**
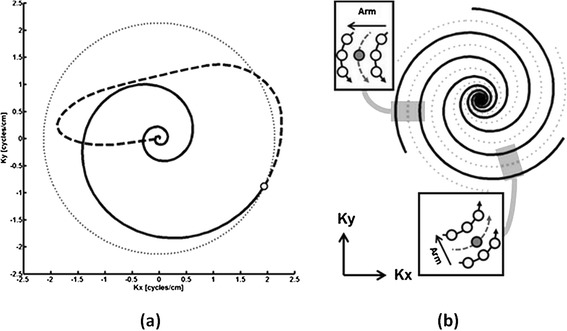
**Ideal spiral trajectory and spiral GRAPPA kernels. (a)** The trajectory for arm 1 (solid curve) reaches the designed edge of k-space (dotted circle) at ~1.7 ms (white dot) from the start of the readout. Data acquired while rewinding gradient moments (dashed curve) were discarded during reconstruction. **(b)** The skipped arms of the dynamic data (dotted gray curves) per partition encoding step were reconstructed with through-time spiral GRAPPA. With respect to increasing sampling time (closed arrowhead) and arm index (open arrowhead) in the two insets, the direction of undersampling as well the relative spacing of known (white circles) and target (gray circles) points of the spiral GRAPPA kernel changes as a function of k-space location.

For the 3D method, the dynamic and calibration volumes were acquired with a 3D stack of spirals bSSFP sequence. The spiral trajectory for in-plane encoding was generated with code developed by Dr. Brian Hargreaves [23]. The design parameters for this trajectory were similar to those used for real-time CMR with 2D though-time spiral GRAPPA [20]. Four arms were used to sample the center 8×8 region of k-space, and a total of 48 arms were used to cover the 128^2^ matrix at the Nyquist sampling rate for FOV 316×316 mm^2^, leading to a spatial resolution of 2.47×2.47 mm^2^. This in-plane resolution is within the guidelines for adult imaging with cine bSSFP (see Table two in [24]). The entire spiral readout required about 2.5 ms of the time between RF pulses. This design (Figure 1a) rewinds the zeroth and first moments of the spiral waveform as suggested by Nayak et al. [25]; however, each acquired arm was truncated prior to reconstruction to the point where the trajectory first hits the k-space excursion that corresponds to the designed image resolution (Figure 1a, white dot).

The slice position and orientation for the 3D spiral scans matched the 2D standard clinical sequence. The key sequence parameters for the 3D scan were: RF pulse duration 1000 μs and time-bandwidth 8; 12 partitions at thickness 8 mm plus 33% oversampling; encoded FOV 316×316 mm^2^, matrix 128×128, readout bandwidth 1563Hz/pixel, minimum TR 4.4 ms. The flip angle ranged from 20° to 47° across this set of volunteers due to SAR constraints. For the ECG-gated 3D cine dynamic acquisition, only 8 of the 48 interleaves in the trajectory were acquired per partition encoding step (in-plane acceleration of R = 6). This yielded a temporal resolution of 35 ms per cardiac phase, where the partition encoding step was incremented with each ECG trigger event. Arrhythmia rejection for prospective triggering was not enabled for this sequence, where the acquisition window for the dynamic 3D spiral scan was set to be 50 ms less than the shortest observed RR interval while acquiring localizer scans per subject. Note that the breathhold duration depended both on the subject’s heart rate and the total number of partitions that were encoded. For the subjects scanned in this study, the breathholds ranged from 10 s to 16 s. In addition to the undersampled dynamic data, 10 repetitions of an ungated, free breathing, fully-sampled stack of spirals acquisition with all 48 arms in each partition using the same 3D spiral sequence parameters served as the calibration data. The total time (including dynamic and calibration scans) was recorded.

### Reconstruction

**Figure 2:**
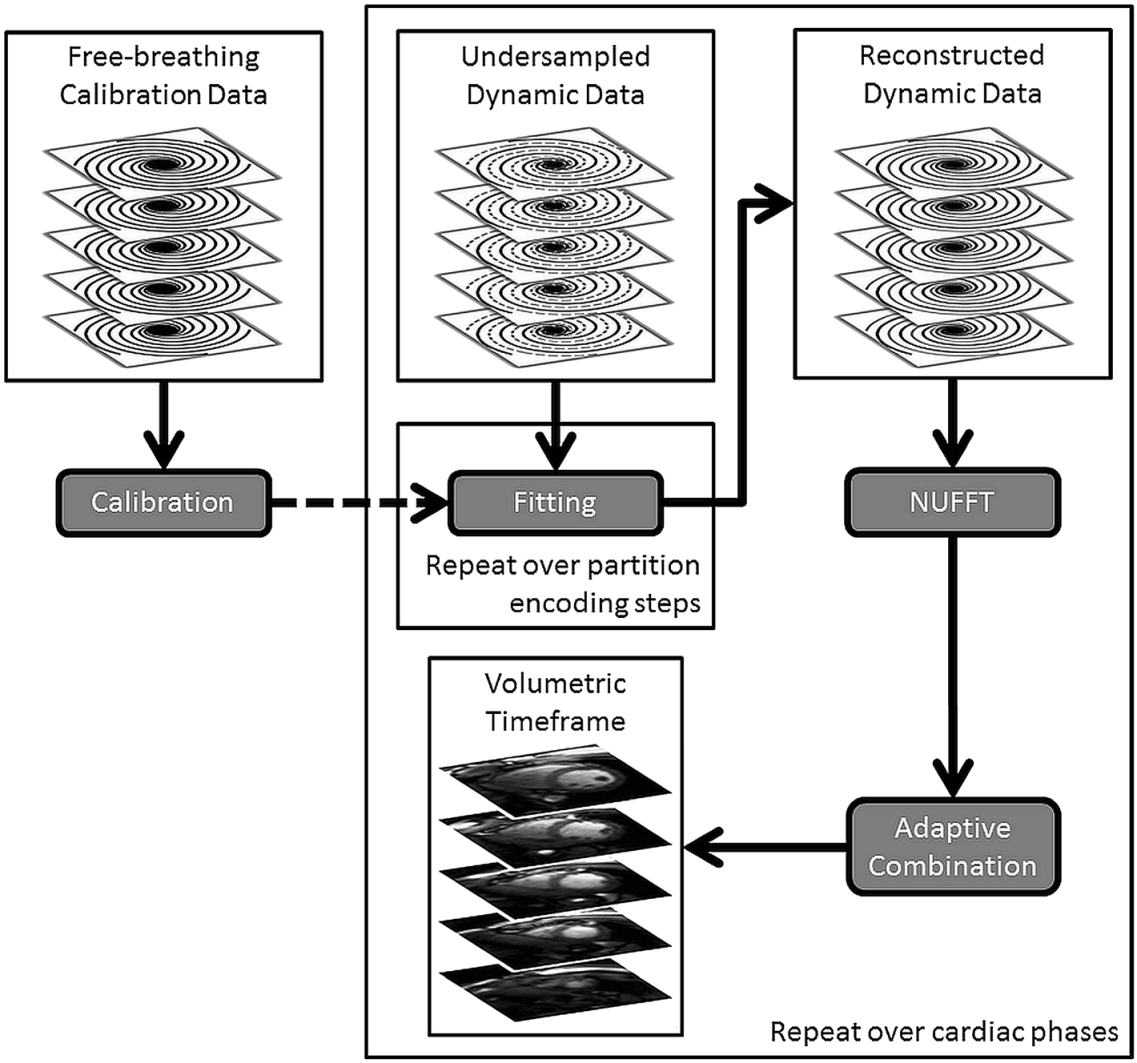
**3D though-time spiral GRAPPA reconstruction flow diagram.** Fully-sampled free-breathing data is used to calibrate the spiral GRAPPA kernels. For each cardiac phase, the skipped arms of the undersampled dynamic data (dashed gray curves) within each partition encoding step were reconstructed with the spiral GRAPPA weights. After NUFFT of the reconstructed dynamic data, the Cartesian coil images were combined with an adaptive algorithm to yield each volumetric timeframe.

The 3D through-time radial GRAPPA [26,27] reconstruction code was modified to support the 3D stack of spirals trajectory. For angularly-undersampled dynamic cine data, the curvilinear shape and direction of acceleration for the local spiral GRAPPA kernel will change as a function of k-space location (Figure 1b), where each missing datum received its own non-Cartesian GRAPPA kernel. The calculation of each set of coefficients exploited three sources of information within the free-breathing calibration data. First, exact replicas of the local sampling pattern were extracted from multiple repetitions of the fully-sampled trajectory [20]. Second, the geometry of the kernel was presumed similar over small regions of k-space to allow segmentation of the trajectory [28]. Third, the temporal footprint of the kernel within a partition encoding step only spans a few RF pulses, such that the partition encoding direction served as yet another independent source of calibration data for a given kernel [26]. In other words, the calibration stage of the reconstruction process (Figure 2) used the so-called through-time, through-k-space and through-volume directions of the calibration data.

All 10 repetitions of fully-sampled free-breathing calibration data [19] and all 16 encoded partitions [21,26] were combined with 1×8 trajectory segmentation [20] to calibrate a 32×2×3 (channel × arm × readout) kernel of spiral GRAPPA coefficients per missing datum. Recall that consecutive arms in the direction of R = 6 acceleration (i.e. 1, 7, 13…) were separated by 6*TR, or about 26 ms, in the calibration data in this study. The heart can be presumed to be quasi-stationary on that timescale. As in 3D through-time radial GRAPPA [21], each kernel of calibrated spiral GRAPPA coefficients was re-used at exactly the same spiral sampling location within each partition encoding step and timeframe. Then the Non-Uniform FFT [29] was used to generate single-coil images from the 3D through-time spiral GRAPPA output, and coil images were combined with an adaptive algorithm [30] with image normalization [31] to yield the volumetric timeframes for calculation of ejection fraction and image rating.

### Systolic left ventricular functional parameter calculation

The images from the standard clinical sequence and the 3D spiral method were manually segmented with vendor-provided software (Argus, Siemens Medical Solutions). This software also extracted end-diastolic volume (EDV), end-systolic volume (ESV), ejection fraction (EF) and end diastolic mass (EDM) from the contours.

### Image rating

An image rating experiment was performed, where two radiologists with 8 and 5 years of CMR experience, respectively, were each presented with the 2D standard clinical or the 3D spiral images in random order. For each presentation, the following items were evaluated on a 5-point Likert scale (1: non-diagnostic, 2: poor, 3: average, 4: good, 5: excellent): level of artifacts; blood-myocardium contrast; sharpness of the endocardial border; and temporal dynamics of the papillary muscles and the left ventricular wall. In this study, a score of 3 (i.e. average) was the minimum threshold for clinical acceptance, where an image rating score of 2 (i.e. poor) or below indicated that the diagnosis could be compromised.

#### Statistical analyses

The difference in mean total scan time across subjects between the 2D and 3D methods was tested for significance with the Welch’s t-test at the p < 0.05 level. A Bland-Altman plot [32] was constructed to identify cases where the observed difference in functional parameter value between imaging methods falls beyond its respective 95% confidence interval. For each case that indicated an outlier, the images were reformatted [33] to the horizontal long-axis view to confirm the presence of slice misregistration in the 2D multi-breathhold data. The observed bias between acquisition methods was tested for significant difference from zero error with a t-test at the p < 0.05 level. The raw scores for each image feature from the two raters were concatenated and compared with one qualitative and one quantitative method. The mean and standard deviation was calculated to provide only a qualitative estimate of central tendency per acquisition method. Differences in image rating between acquisition methods were evaluated with a Wilcoxon signed-rank test [34] using the concatenated scores. The null hypothesis was that the set of observed score differences (i.e. 3D-2D) was sampled from a distribution with a median of zero, where significant difference was set at the p < 0.05 level.

## Results

**Figure 3:**
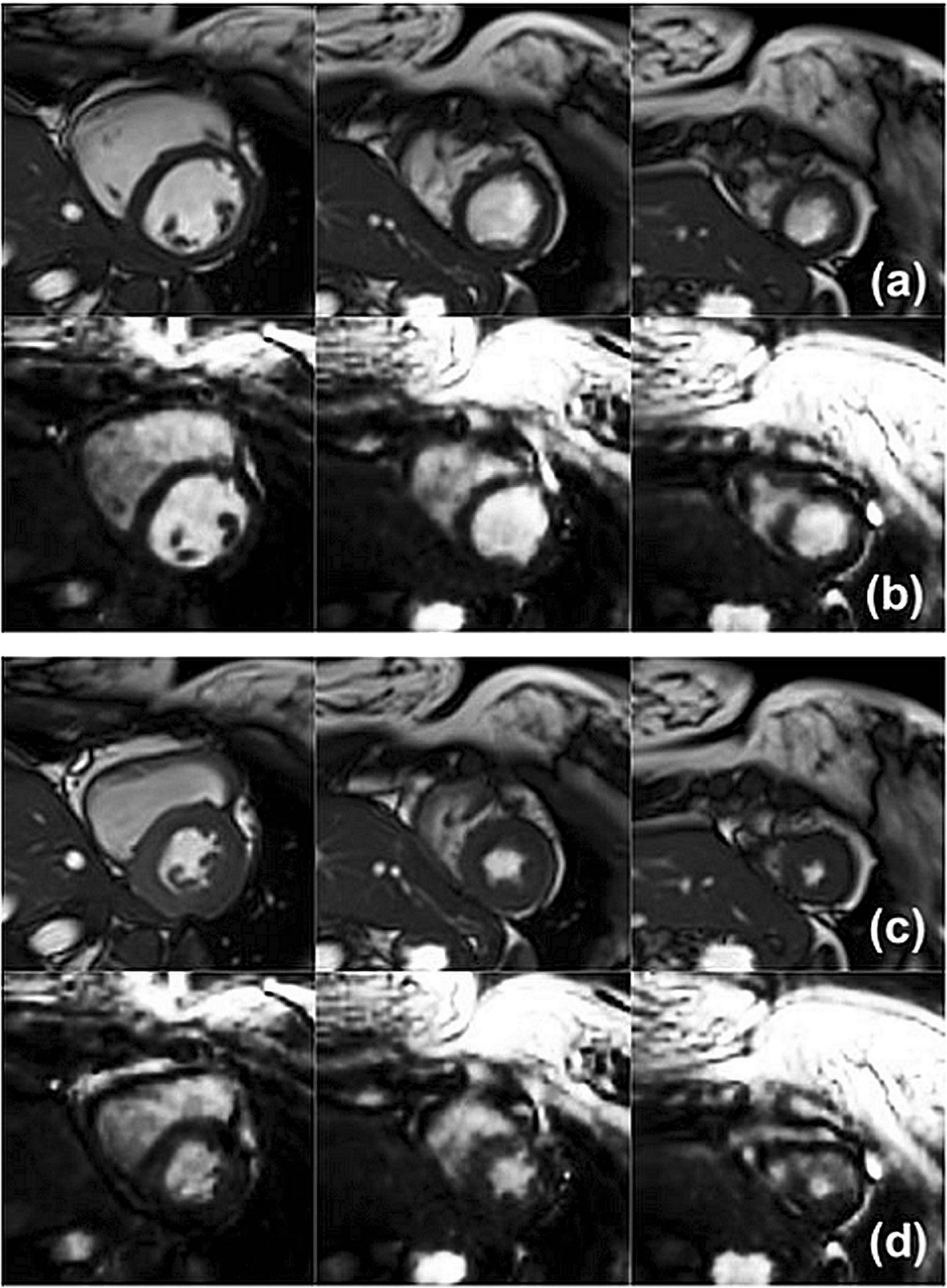
**Timeframes for manual segmentation.** Basal, mid and apical slice planes (left to right) are shown at end-diastole **(a,b)** and end-systole **(c,d)** for 2D **(a,c)** and 3D **(b,d)** scans. Images were cropped to 150 mm x 150 mm about the myocardium.

**Figure 4:**
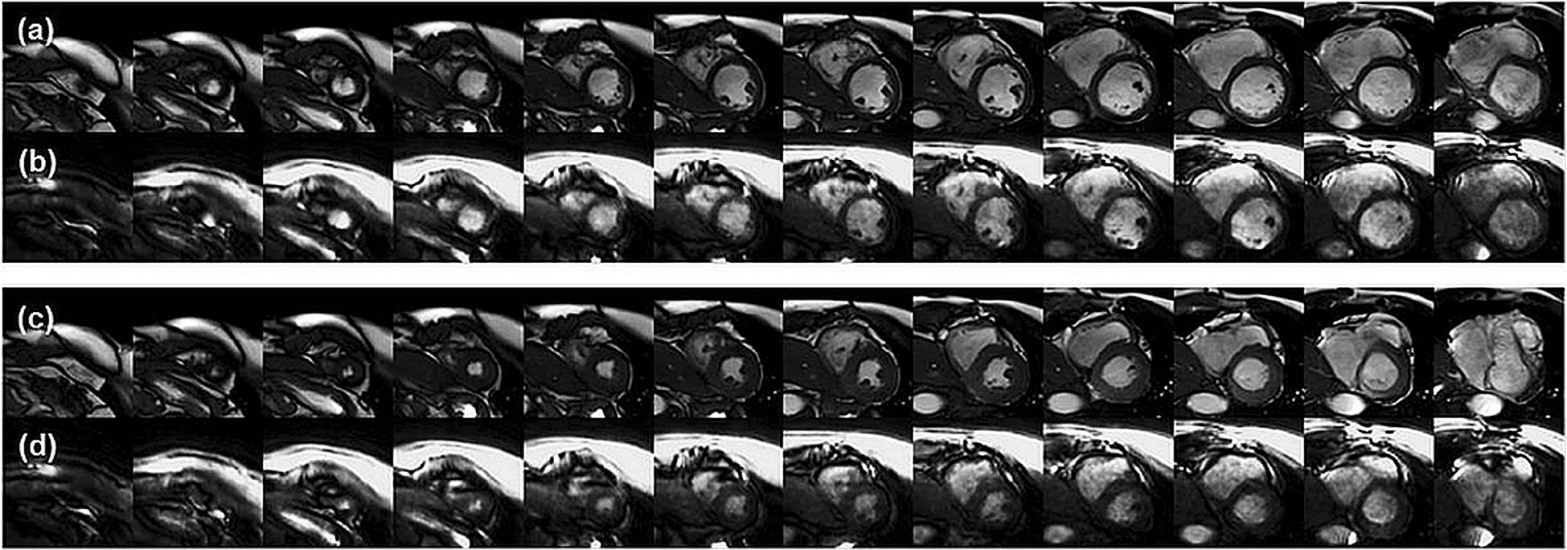
**Left ventricular coverage.** Twelve short-axis slice planes from apex to base (left to right) at end-diastole **(a,b)** and end-systole **(c,d)** for 2D **(a,c)** and 3D **(b,d)** scans are shown. Images were cropped to 150 mm x 150 mm about the myocardium.

Representative image reconstruction results are shown in Figures 3 and 4. The respective scaling was independently adjusted to yield similarly bright blood pool and similarly dark myocardium to facilitate comparison between the 2D and 3D images methods in these figures. The images were cropped to a FOV of 150×150 mm^2^ to focus on the myocardium. Figure 3 shows example basal, mid and apical slice planes from left to right at end-diastole (Figure 3a,b) and end-systole (Figure 3c,d). The sharpness of the border in this case was scored as excellent by both raters for the 2D scan, and the 3D scan was scored as good by rater 1 and excellent by rater 2. Figure 4 demonstrates left ventricular coverage and image contrast for the 2D (Figure 4a,c) and 3D (Figure 4b,d) scans. The blood-myocardium contrast in this case was scored as excellent by both raters for the 2D scan, and the 3D scan was scored as average by rater 1 and good by rater 2. In order to aid visualization of image quality differences, corresponding movies of 2D and 3D cine results have been provided as Additional files 1,2,3,4.

**Figure 5:**
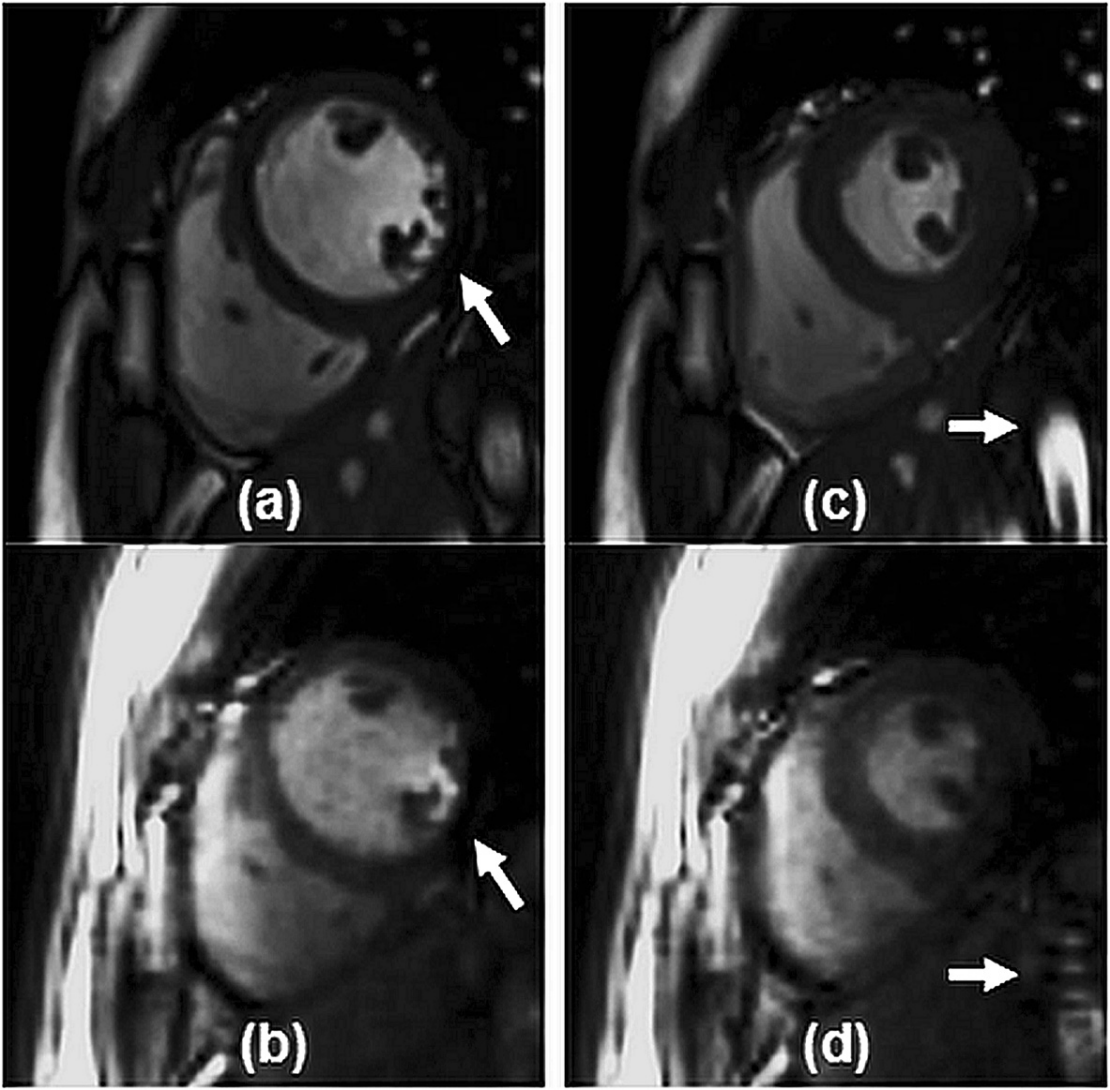
**Visual appearance of artifacts.** Differences between 2D **(a,c)** and 3D **(b,d)** imaging techniques are demonstrated for off-resonance effects **(a,b)** and through-plane flow in the descending aorta during systole **(c,d)**. Images were cropped to 150 mm x 150 mm about the myocardium.

Figure 5 highlights differences between the imaging methods with respect to artifacts. At end-diastole, off-resonance banding was not observed for the 2D scan (Figure 5a, arrow). A banding artifact in the myocardium with brightening of adjacent blood pool pixels (Figure 5b, arrow) was observed for the 3D scan at a similar slice plane. During systole, flow artifacts from accelerating blood in the descending aorta were oriented along a single phase encoding direction for the 2D scan (Figure 5b, arrow), but this appeared as a local and short-lived swirling artifact in the 3D scan (Figure 5c, arrow). Note that artifacts for this volunteer were scored as excellent (meaning little to no artifacts) by both raters, and the 3D scan was scored as good by both raters. Banding and flow artifacts can also be appreciated in movies of the 2D and 3D cine results, which can be found in Additional files 5 and 6, respectively.

**Figure 6:**
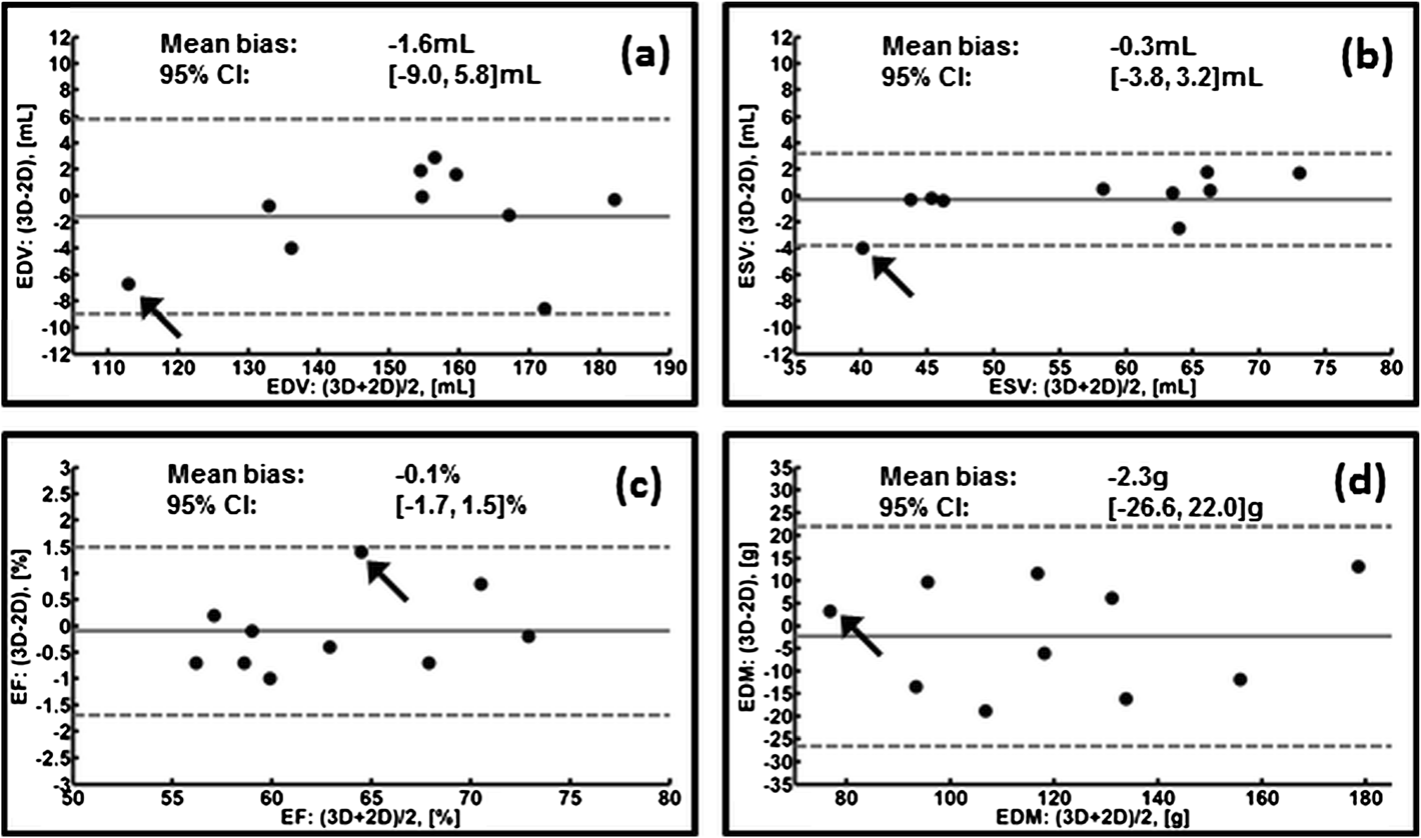
**Bland-Altman plots for left ventricular functional values.** For 3D vs. 2D values, **(a)** end-diastolic volume (EDV), **(b)** end-systolic volume (ESV), **(c)** ejection fraction (EF), and **(d)** end diastolic mass (EDM) plots are shown. The gray horizontal lines in each panel are the mean bias (solid) and the 95% confidence interval (dashed). Arrows highlight one case, where only the ESV difference is slightly beyond its respective confidence interval (−4.0 mL vs. -3.8 mL lower limit).

**Figure 7:**
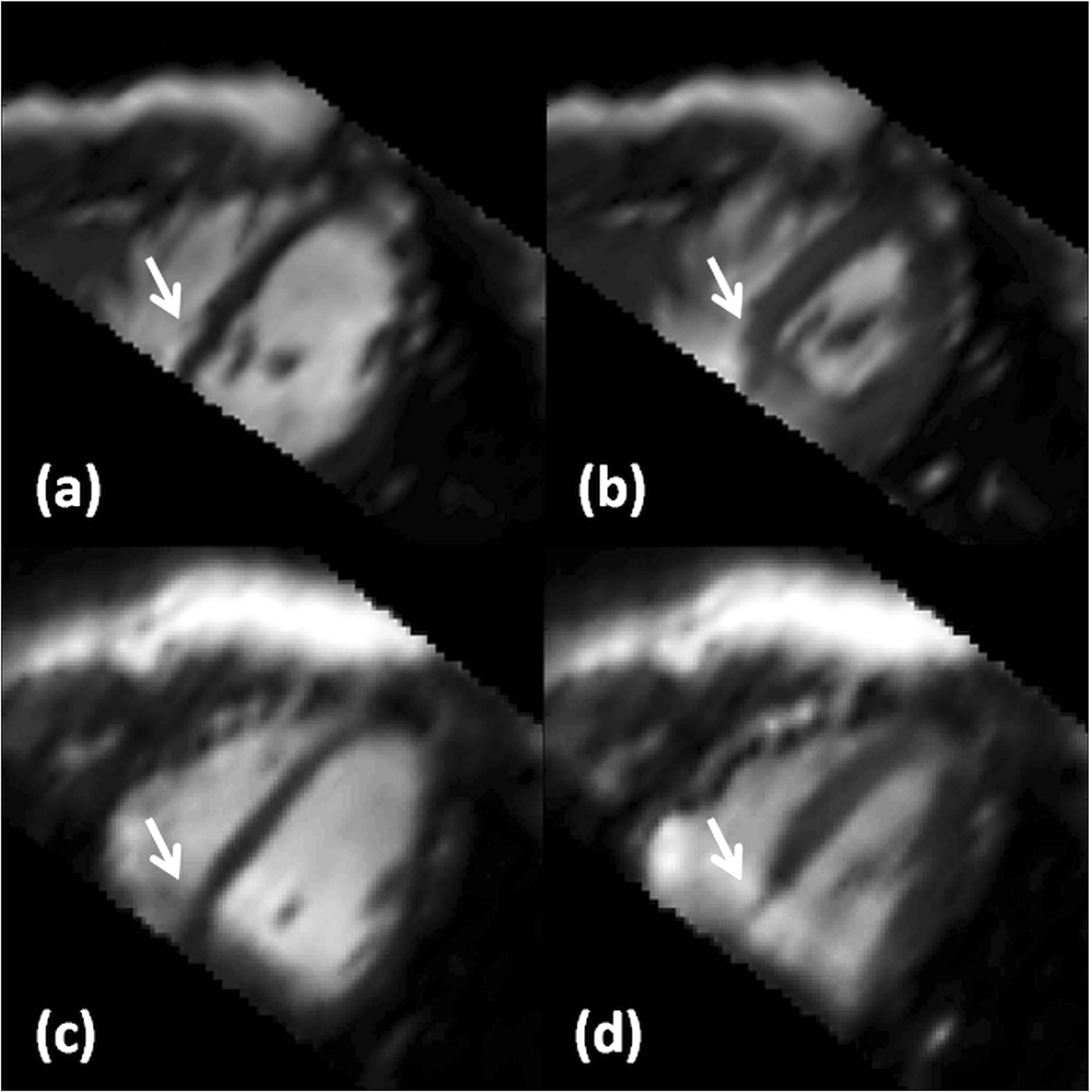
**Reformatted views.** For one case with an ESV outlier in the Bland-Altman plot (Figure 6, arrows), the horizontal long axis view for 2D **(a,b)** and 3D spiral **(c,d)** is shown after reformatting from the acquired short-axis view for the **(a,c)** end-diastolic and **(b,d)** end-systolic timeframes. Arrows highlight similar basal slice planes for both imaging techniques, where slice misregistration for 2D imaging can be observed.

Figure 6 shows a set of Bland-Altman plots, where the difference (3D-2D) was plotted against the mean of each pair of systolic left ventricular functional values. The mean (Figure 6, solid gray line), standard deviation and 95% confidence interval (Figure 6, dashed gray lines) for the bias between the two scan types for the respective Bland-Altman plot has been summarized in Table 1. Only one case shows end-systolic volume difference beyond its respective 95% confidence interval for this case (Figure 6b, arrow). End-diastolic volume (Figure 6a, arrow), ejection fraction (Figure 6c, arrow) and stroke volume (not shown) – were within their limits. After reformatting from short-axis to horizontal long axis, misregistration of the basal slice planes was evident for the 2D multi-breathhold data (Figure 7a,b arrow; Additional file 7) with respect to the single-breathhold 3D spiral result (Figure 7c,d; Additional file 8). The 3D method underestimated end-diastolic mass with respect to the 2D method by 2.3 g (Table 1). All differences in end-diastolic mass between the imaging techniques fall within the 95% confidence interval of [−26.5, 22.0]g, including the case with the end-systolic volume outlier (Figure 6d, arrow). For the functional parameters examined in this study, the bias between imaging methods was not significantly different from zero (t-test, p > 0.05, Table 1).

**Table 1:** **Summary of Bland-Altman analysis**

**Bland-altman statistic**	**Functional metric**			
	**EDV [mL]**	**ESV [mL]**	**EF [%]**	**Mass [g]**
Bias	−1.6	−0.3	−0.1	−2.3
SD of Bias	3.8	1.8	0.8	12.4
Min. Limit (95%)	−9.0	−3.8	−1.7	−26.6
Max. Limit (95%)	5.8	3.2	1.5	22.0
p-value	0.19	0.62	0.60	0.57

The 3D spiral acquisition demonstrated statistically significant savings in total scan time. The 2D multi-breathhold sequence required 340 ± 40 s (mean ± SD) to collect the set of 12 slices. The 3D spiral dynamic scan required a single breathhold of 14 ± 2 s (mean ± SD) plus a fixed time of 34 s under free-breathing conditions to acquire the calibration data for the 3D through-time spiral GRAPPA reconstruction. The Welch’s t-test, which accounts for samples with uneven variance (as well as size), yielded a t-value of 22.9 with degrees of freedom 9; this corresponds to a significant difference in total scan time between the methods with p = 2.7e-9.

**Figure 8:**
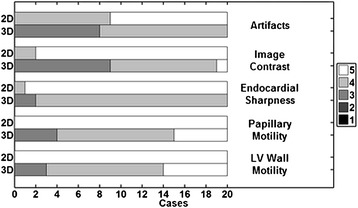
**Image rating scores.** The pooled scores from both raters per imaging method are shown as stacked horizontal bar graphs for the noted image feature. The relative scoring (3D-2D) for all image features was significantly different (Wilcoxon signed-rank test, p < 0.05). Table 2 summarizes the outcomes of the image rating study.

The raw image scores for both image raters are compiled in Figure 8, and the descriptive statistics have been summarized in Table 2. Although the relative scaling between the 2D and 3D images in Figures 3 and 4 focused on blood-myocardium contrast at the expense of the chest wall, the image raters could freely adjust the display of DICOM image series from each method according to the their clinical experience and preference. For all image features that were analyzed, the mean score across subjects was consistently lower for 3D imaging. The Wilcoxon signed-rank test showed significant differences (p < <0.05) between the 3D spiral and 2D standard scan for all image features.

**Table 2:** **Image rating results**

**Image feature**	**2D**	**3D**	**pWSR**
Artifacts	4.6 ± 0.5	3.6 ± 0.5	1.2E-04
Image contrast	4.9 ± 0.3	3.6 ± 0.6	1.2E-04
Endocardial sharpness	5.0 ± 0.2	3.9 ± 0.3	2.9E-05
Papillary muscle motility	5.0 ± 0.0	4.1 ± 0.7	6.1E-05
LV wall motility	5.0 ± 0.0	4.2 ± 0.7	1.2E-04

Mean ± SD across all subjects after pooling scores from both raters.

Significant difference for Wilcoxon signed rank test was set at p_WSR_ < 0.05.

## Discussion

This study describes a method for rapidly acquiring volumetric timeframes of left ventricular motion for extracting functional parameter values. This method encoded angularly-undersampled ECG-gated dynamic data in one breathhold with a 3D stack of spirals trajectory, and collected calibration data under free-breathing conditions to perform offline 3D through-time spiral GRAPPA. Note that patients with severe arrhythmia would be contraindicated from multi-slice imaging as well as this rapid 3D spiral technique, as prospectively gated dynamic imaging presumes a normal sinus rhythm. Nonetheless, in this study of healthy volunteers, imaging and analysis could be performed in all ten cases.

Systolic left ventricular functional parameter differences between the 3D spiral and 2D imaging methods were not significant (t-test, p > 0.05, Table 1). Reformatting the one case with an ESV difference outlier (Figure 6 arrows) identified misregistration in the basal slices for 2D multi-breathhold imaging (Figure 7a,b, arrow; Additional file 7) with respect to single-breathhold 3D spiral imaging (Figure 7b,d; Additional file 8). Variable diaphragm position, which affected repeatability studies [4] and has been demonstrated for other 3D cardiac cine imaging strategies [15,16], may have contributed to the discrepancy in ESV between imaging methods. The LV ejection fraction in this sample of healthy volunteers was 62.9 ± 6.0% and 63.0 ± 5.7% (mean ± SD) for 3D spiral and 2D imaging, respectively. These values are in good agreement with a similar comparison in a report of 3D radial imaging in 10 healthy volunteers (see LVEF in Table 2 in Peters, et al. [15]). With respect to LV end-diastolic mass, the standard deviation of the bias between 3D spiral and 2D imaging methods in this study was 12.4 g (Table 1), whereas 3D radial imaging reported 6.5 g (as per LVEDM in Table 2 in Peters, et al. [15]). For context, note that the average change in myocardial mass due to treatment of hypertrophic cardiomyopathy with ACE inhibitors over one year was reported as -45 g as measured by echocardiography [35–37]. However, as noted for 3D radial imaging [15], improvements to image contrast between the lung and the myocardium for 3D spiral imaging may improve conspicuity of the epicardial border and reduce the variability in measurements of myocardial mass with respect to 2D imaging.

Although the average total scan time for 3D spiral imaging was significantly shorter than the 2D multi-breathhold standard of this study, the results will vary with pulse sequence and reconstruction parameters. Within respect to a 16RR breathhold for 3D spiral imaging, 2D multi-slice imaging with the listed parameters could have supported the encoding of two slices per 14RR breathhold. Despite conceding that the scan time advantage from our results would have been halved at low risk to multi-slice image quality in healthy volunteers, the 3D spiral method would still be significantly faster. Reducing the in-plane resolution and increasing the acceleration factor of the 2D sequence could have shortened the total scan time for the reference method as well. Low resolution 2D images with R = 4 could enable the collection of up to five slices per breathhold, leading to total acquisition times on the order of the 3D spiral method, albeit with more breathholds. However, the clinically-relevant 2D gold-standard is a high resolution, low acceleration factor acquisition. On the other hand, strategies to reduce the data collection time for 3D spiral could be used. In the original 3D through-time GRAPPA publication [21], acceptable image quality for renal angiography was observed using just two fully-sampled datasets for calibration [21]. For the 3D spiral trajectory of this study (48 arms and 16 partitions including oversampling at TR 4.4 ms), the total scan time to collect just two repetitions would be roughly 7 s. As in the analyses of the 2D [19] and 3D radial [21] methods, simulation studies of 3D through-time spiral GRAPPA for cardiac cine imaging are expected to support increasing the trajectory segmentation to account for fewer repetitions of calibration data in an optimized acquisition. Furthermore, the partition encoding direction of the 3D spiral dynamic scan can be accelerated with combinations of partial Fourier acceleration and through-time non-Cartesian GRAPPA [38,39] to shorten the breathhold, increase partition encoding resolution or expand the volumetric coverage. In the end, conservative scan and reconstruction parameters were selected for both methods at the expense of scan efficiency, where multiple slices with R = 2 [12], one slice with R = 1 [14,22] and one slice with R = 2 (as used in this study) per breathhold all yield high quality images against which ventricular functional parameters and image quality of a test method can be compared.

The Wilcoxon signed-rank test results (Table 2) showed significant differences in qualitative scoring for all image features, where the 2D images were favored for several reasons. Balanced SSFP off-resonance effects were greater for the 3D spiral scan (Figure 5b, arrow) due to a longer TR (4.4 ms) than the 2D sequence (3.26 ms) as well as a reduced flip angle (average 29°) due to SAR constraints. With respect to image contrast, reduced blood signal enhancement from in-flow of unexcited blood has been shown for 3D cine cardiac imaging relative to a 2D scan [13]. Furthermore, lower image contrast for 3D spiral imaging in general, may have contributed to challenging conditions for drawing the epicardial contour as shown in prior studies [15]. If left ventricular functional imaging follows a contrast-enhanced perfusion or an arterial input function scan, substantial improvements to the blood-myocardium and lung-myocardium contrast can be expected for the 3D spiral method [10,40,41] with a corresponding reduction in the 95% confidence interval for differences with non-enhanced 2D imaging (see Figure two in [41]). With respect to endocardial sharpness, the difference in spatial resolution relative to 2D imaging contributed to lower scores for the 3D spiral method, yet significant differences in ventricular volume measurements at end-systole and end-diastole were not observed (Figure 6). With respect to lower motility scores for the 3D spiral method, some cases demonstrated reduced but still acceptable image quality in the most basal plane in comparison to the remainder of the 3D volume (Figure 4b,d). Unexpected shifting of anatomy relative to the planned imaging volume was suspected, where respiratory bellows might be useful in future studies to gauge the contribution of breathhold position to observed variations between imaging methods. Although patients were not included in this study, the difference of in-plane resolution between 3D spiral and 2D imaging is not expected to significantly affect qualitative diagnosis of regional abnormalities in left ventricular wall thickening.

Although the 3D spiral images yielded equivalent systolic LV functional parameters in less total scan time than 2D multi-slice imaging with image rating scores of good or better, there are some limitations to this study to consider. First, for imaging pediatric patients [24] or the unusual case of detecting a subtle wall motion abnormality, 2.5 mm spatial resolution for 3D spiral imaging may be too low despite temporal resolutions under 40 ms per cardiac phase. Spiral readout waveforms with 1.63 mm spatial resolution can be designed for bSSFP; however, the minimum TR will be greater than 4.4 ms, such that off-resonance artifacts may become more pronounced, and in-plane acceleration may need to be increased to maintain high temporal resolution for the dynamic scan. Second, one case (Figure 5b,c) demonstrated mild off-resonance artifacts despite second order shimming over the volume of the heart. Note that dark bands were located at 227 Hz intervals in this study. This is much larger than 125 Hz, or the average range for off-resonance using the same shimming method as reported by other investigators on a 3 T Skyra system [42]. However, the same typical range is only slightly less than 151 Hz, which considers a 33% safety margin [43] for off-resonance across the cardiac anatomy. Other shimming methods [44,45] may further decrease the expected range of off-resonance to enable robust 3D spiral imaging with even longer TR values to match the same high in-plane resolution as a 2D Cartesian standard. Third, the bright pixels in the anterior chest wall of the 3D spiral images and movies suggest that applying the normalization method [31] to non-Cartesian reconstruction may need to consider additional factors. For example, pixels in the anterior chest wall can contain gridding alias in channel images obtained from the posterior elements of the receiver array for the 3D spiral acquisition. This may have violated the presumption of a stochastic random process between signal and noise across channels for these pixels. Although using a body coil reference image or a non-parametric method [46] may be more robust, the analysis of the cardiac anatomy and its function in this study was not affected by the bright chest wall. Fourth, the contrast difference between 3D spiral and 2D imaging was only indirectly quantified with an analysis of subjective scoring by radiologists. Using pixel values within regions of interest, as shown in other studies [9,15,41], should likewise show equivalent ventricular functional parameter values to 2D imaging despite a significant decrease in blood-myocardium contrast for 3D imaging. However, quantifying SNR and CNR may be more important when evaluating strategies to improve image contrast for 3D spiral imaging. Finally, this study was not designed to quantify the technical performance or improve the reconstruction speed of the 3D through-time spiral GRAPPA method itself. Many technical details of 3D through-time non-Cartesian GRAPPA are discussed in [21]. Note that offline reconstruction time for 3D through-time spiral GRAPPA using non-optimized Matlab code required 82 ± 8 min (mean ± SD) for the cases in this study. Early unpublished results indicate that the entire reconstruction can be performed in about 7 min for an online Central Processing Unit (CPU) method. As shown for quantitative renal perfusion [47], the total reconstruction time for 3D spiral data in this study is estimated to be less than 60 s for an offline Graphics Processing Unit (GPU) implementation. However, as initially demonstrated for real-time cardiac imaging [48,49], calibration and reconstruction durations could become a negligible component of total scan time for a low-latency online GPU implementation of 3D through-time spiral GRAPPA. The limitations of this study and optimizations to 3D through-time spiral GRAPPA can be addressed in future studies, where using just one breathhold for dynamic imaging is anticipated to reduce patient fatigue as part of the complete cardiac examination.

## Conclusions

The 3D through-time spiral GRAPPA method demonstrated equivalent left ventricular functional parameter values and required significantly less total scan time than 2D segmented multi-breathhold cine imaging in this study. Furthermore, 3D spiral image quality was acceptable and did not hinder the calculation of systolic left ventricular functional parameters.

## Additional files

Additional file 1:
**First movie of 2D cine results.** Whereas Figure 3a,c only showed three of the slice locations that were used for manual segmentation at end-systole and end-diastole, this movie shows all 12 acquired slices across the entire cardiac cycle. Note that the source images for each slice were cropped to 150 mm x 150 mm about the myocardium. Additional file 2:
**First movie of 3D spiral cine results.** Although Figure 3b,d only showed three of the partitions that were used for manual segmentation at end-systole and end-diastole, this movie shows the contiguous block of the central 12 partitions that corresponded to the planned coverage for 2D imaging (i.e. Figure 3a,c and Additional file 1). Note that the source images for each partition were cropped to 150 mm x 150 mm about the myocardium. Additional file 3:
**Second movie of 2D cine results.** The left ventricular coverage of 2D imaging in a second volunteer as shown in Figure 4a,c can be appreciated across the cardiac cycle in this movie. The source images for each slice were cropped to 150 mm × 150 mm about the myocardium. Additional file 4:
**Second movie of 3D spiral cine results.** The left ventricular coverage of 3D spiral imaging in a second volunteer as shown in Figure 4b,d can be appreciated across the cardiac cycle in this movie with respect to the planned coverage for 2D imaging (i.e. Figure 4a,c and Additional file 2). Note that the source images for each partition were cropped to 150 mm x 150 mm about the myocardium. Additional file 5:
**Third movie of 2D cine results.** The banding (or lack thereof) and flow artifact for 2D imaging in a third volunteer as noted in Figure 5a,c can be appreciated across the cardiac cycle in this movie of one slice location. The source images were cropped to 150 mm × 150 mm about the myocardium. Additional file 6:
**Third movie of 3D spiral cine results.** The change in banding and flow artifact for 3D spiral (Figure 5b,d) relative to 2D imaging (Figure 5a,c, Additional file 5) in a third volunteer can be appreciated across the cardiac cycle in this movie. The planned partition that most closely approximated the acquired 2D slice location has been shown here. The source images were cropped to 150 mm × 150 mm about the myocardium. Additional file 7:
**Reformatted 2D cine results.** For the case with an ESV difference outlier (Figure 6, arrows), the 2D cine images were reformatted, where the slice misregistration as noted in Figure 7a,b can be appreciated across the cardiac cycle in this movie. The source images were cropped to 150 mm × 150 mm about the myocardium. Additional file 8:
**Reformatted 3D spiral cine results.** For the case with an ESV difference outlier (Figure 6, arrows), the 3D cine images were reformatted. Whereas slice misregistration for the reformatted 2D cine images as noted in Figure 7a,b, the contiguous coverage of the left ventricle as shown in Figure 7c,d can be appreciated across the cardiac cycle in this movie. The source images were cropped to 150 mm × 150 mm about the myocardium.

## Abbreviations

bSSFP: Balanced steady State with free precession
CMR: Cardiovascular magnetic resonance
ECG: Electrocardiogram
FOV: Field-of-view
GRAPPA: Generalized autocalibrating partially parallel acquisitions
LV: Left ventricle
NUFFT: Non-uniform fast fourier transform
SENSE: Sensitivity encoding
SD: Standard deviation
